# Synthesis, solid-state fluorescence properties, and computational analysis of novel 2-aminobenzo[4,5]thieno[3,2-*d*]pyrimidine 5,5-dioxides

**DOI:** 10.3762/bjoc.8.28

**Published:** 2012-02-16

**Authors:** Kenichirou Yokota, Masayori Hagimori, Naoko Mizuyama, Yasuhisa Nishimura, Hiroshi Fujito, Yasuhiro Shigemitsu, Yoshinori Tominaga

**Affiliations:** 1 Faculty of Environmental Studies, Nagasaki University, 1-14, Bunkyo-machi, Nagasaki 852-8521, Japan; 2 Faculty of Pharmaceutical Sciences, Nagasaki International University, 2825-7, Huis Ten Bosch, Sasebo 859-3298, Japan; 3 Department of Pharmacy, Saga University Hospital, 5-1-1, Nabeshima, Saga 849-8521, Japan; 4 Industrial Technology Center of Nagasaki, Omura, Nagasaki, 856-0026, Japan

**Keywords:** amino group, fluorescence, HOMO/LUMO, pyrimidine, solid-state

## Abstract

New fluorescent compounds, benzo[4,5]thieno[3,2-*d*]pyrimidine 5,5-dioxides (**3a–g**), 2-amino-4-methylsulfanylbenzo[4,5]thieno[3,2-*d*]pyrimidine (**6**), and 2-amino-4-methylsulfanyl-7-methoxybenzo[4,5]furo[3,2-*d*]pyrimidine (**7**), were synthesized in good yields from heterocyclic ketene dithioacetals (**1a**–**c**) and guanidine carbonate (**2a**) or (*S*)-methylisothiourea sulfate (**2b**) in pyridine under reflux. Among the fused pyrimidine derivatives, compound **3c**, which has an amino group at the 2-position and a benzylamino group at the 4-position of the pyrimidine ring, showed the strongest solid-state fluorescence. The absorption and emission properties of the compounds were quantitatively reproduced by a series of ab initio quantum-chemical calculations.

## Introduction

Solid-state fluorescent compounds are currently attracting considerable interest from both theoretical and practical standpoints [1–4]. Recently, we prepared new pyrimidine derivatives, which have solid-state blue fluorescence, by means of a one-pot synthesis, involving the reaction of ketene dithioacetals, amines, and guanidine carbonate in pyridine [5]. We have also reported the one-pot synthesis of a new, fluorescent, fused pyrimidine derivative 2,4-diaminoindeno[1,2-*d*]pyrimidin-5-one; this pyrimidine derivative, which was synthesized by heating a ketene dithioacetal, 2-[bis(methylsulfanyl)methylidene]indan-1,3-dione, under reflux with amine and amidine derivatives in pyridine solution, showed blue-green fluorescence in the solid state [6]. These one-pot synthetic methods are also promising for the preparation of other new solid-state fluorescent pyrimidine derivatives containing polycyclic heterocycles. In this paper, we report the synthesis of new fluorescent 2-aminobenzo[4,5]thieno[3,2-*d*]pyrimidine 5,5-dioxides and related fused pyrimidine derivatives. It is thought that these derivatives show strong fluorescence as a result of the presence of a hetero-π-electron conjugated system [7]. The electronic and emission spectra of these new compounds were analyzed computationally by using a series of ab initio quantum-chemical calculations.

## Results and Discussion

### Synthesis

**Scheme 1:**
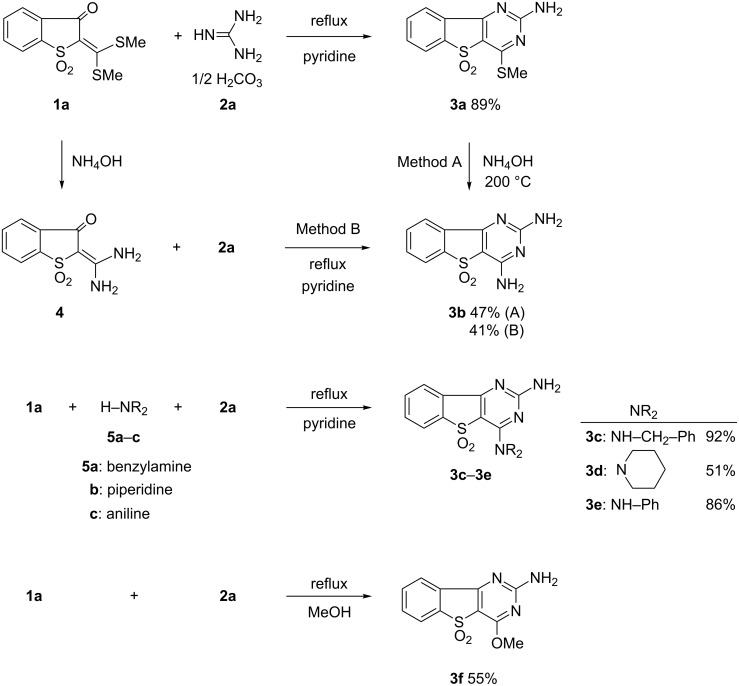
Syntheses of benzo[4,5]thieno[3,2-*d*]pyrimidine 5,5-dioxides (**3a**–**f**).

Heterocyclic ketene dithioacetals (**1a–c**) were easily prepared by the condensation of the corresponding heterocyclic active methylene compounds with carbon disulfide in sodium hydroxide solution as a base, followed by methylation with dimethyl sulfate [8–10]. The reaction of **1a** with guanidine carbonate (**2a**) in pyridine under reflux gave the expected product, 2-amino-4-(methylsulfanyl)benzo[4,5]thieno[3,2-*d*]pyrimidine 5,5-dioxide (**3a**) in 89% yield (Scheme 1). Based on our findings that diaminopyrimidine derivatives show blue fluorescence in the solid state [5], we attempted to synthesize diaminopyrimidine derivatives (**3b**–**e**), expecting them to show intense solid-state fluorescence. 2,4-Diaminobenzo[4,5]thieno[3,2-*d*]pyrimidine 5,5-dioxide (**3b**) was easily obtained, in 47% yield, by the displacement reaction of **3a** with 28% ammonia solution at 200 °C for 3 h in a mini-autoclave. Compound **3b** was also synthesized by the reaction of **2a** with 2-(diaminomethylene)benzo[*b*]thiophene-3(2H)-one 1,1-dioxide (**4**), which was easily obtained from **1a** by using 28% ammonia in methanol under reflux. The reactions of **1a**, **2a**, and amines **5a**–**c** (benzylamine, piperidine, and aniline) in pyridine under reflux gave diaminopyrimidine derivatives **3c**–**e** in good yields, i.e., 92%, 51%, and 86%, respectively. The reaction of **1a** and **2a** under reflux in methanol instead of pyridine gave the methoxy pyrimidine derivative **3f** in 55% yield.

**Scheme 2:**
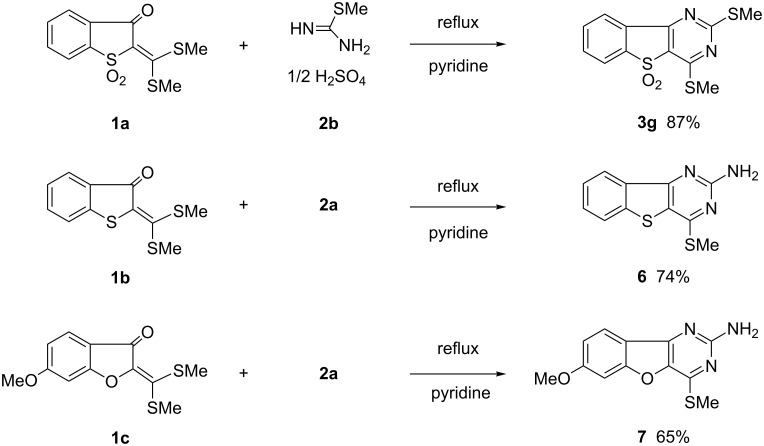
Syntheses of **3g**, **6**, and **7**.

In the presence of sulfuric acid solution, 2,4-dimethylsulfanyl derivative **3g** was obtained from **1a** with (*S*)-methylisothiourea sulfate (**2b**) in 87% yield (Scheme 2). Fused pyrimidine derivatives **6** and **7** containing the electron-rich heterocycles benzothiophene or benzofuran were prepared by the reaction of ketene dithioacetal **1b** or **1c** with guanidine carbonate (**2a**) in 74% and 65% yield, respectively.

### Computational methods

The ground-state (S_0_) geometries were optimized by means of density functional theory (DFT) with the B3LYP hybrid functional and 6-311G(d,p) [11] basis set (DFT(B3LYP)/6-311G(d,p)). The first excited-state (S_1_) geometries were optimized with configuration interaction singles CIS/6-311G(d,p) and the complete active space SCF CASSCF(10,10)/ANO-S-MB [12] level of theory. Time-dependent TDF (TDDFT), CASSCF, and multistate complete active-space second-order perturbation theory (MS-CASPT2) calculations were subsequently performed for the optimized geometries to evaluate the vertical transition energies along with the associated transition characteristics. The TDDFT calculations consistently employed the B3LYP functional and 6-31+G(d,p) basis set [13]. Solvent effects were evaluated by using the linear-response polarized-continuum model for ethanol (PCM(ethanol)-TDDFT(B3LYP)/6-31G+(d,p)) [14]. MS-CASPT2 used ten active electrons distributed within eight active spaces combined with an ANO-S basis set (MS-CASPT2(10,8)/ANO-S). The DFT, CIS, and TDDFT computations were performed by using Gaussian 09 software [15], and CASSCF and MS-CASPT2 were performed by using the MOLCAS 7.4 suite of programs [16].

### Comparison of predicted and experimental absorption spectra

**Figure 1:**
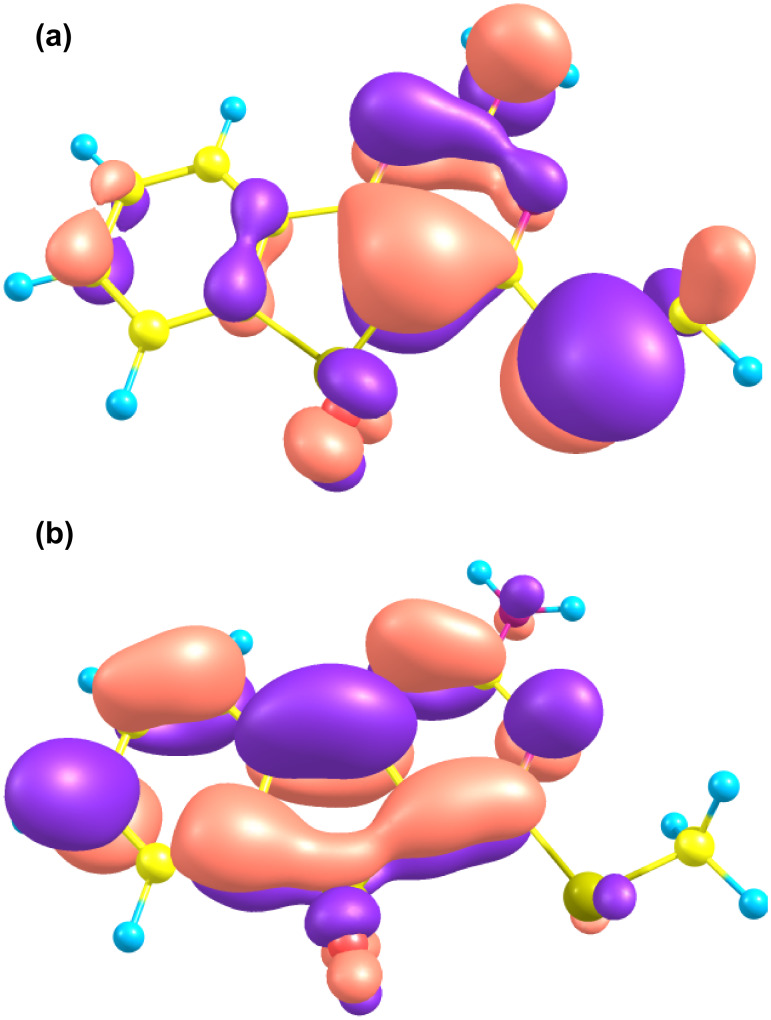
(a) HOMO and (b) LUMO for **3a**, computed with TD-DFT(B3LYP)/6-31+G(d,p). The pink (blue) lobes indicate a positive (negative) isocontour value of 0.03.

**Figure 2:**
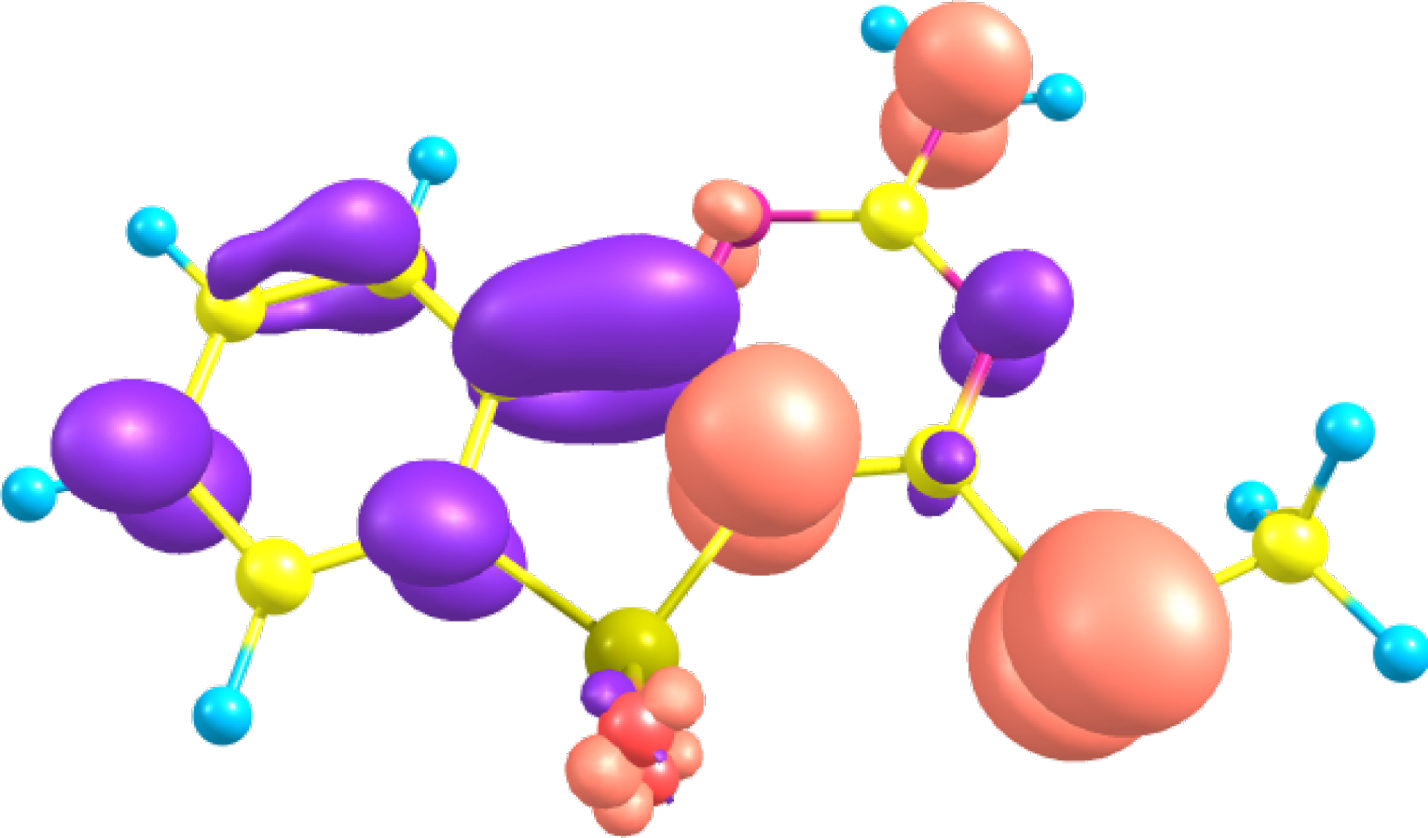
The distribution of the HOMO minus LUMO density for **3a**, computed with TD-DFT(B3LYP)/6-31+G(d,p). The pink (blue) lobes indicate a positive (negative) isocontour value of 0.003, respectively.

The UV–vis spectra of the compounds exhibited peaks (λ_max_) at 280–360 nm. The spectra possessed multiple subpeaks around λ_max_. The experimental and computational λ_max_ values are given in Table 1. The theoretical and experimental λ_max_ values are in fairly good agreement, within 50 nm deviation. The red-shifts observed for **3d**, **3e**, and **6** relative to **3b** were well reproduced by the TDDFT computations. The sidebands around λ_max_ were not reproduced by our TDDFT computations, which predicted single-peak maxima for all the compounds. The subpeaks are considered to be vibronically assisted absorptions, because the extended π-systems of the molecules have large degrees of vibrational freedom effectively coupled with their electronic states. As a representative example of the electronic structures, the HOMO and LUMO of **3a** are depicted in Figure 1. The λ_max_ was assigned as the HOMO–LUMO π–π* excitation (configuration weight = 0.697) with an oscillator strength of 0.096 at 335 nm. The next theoretical peak appeared at 290 nm, derived from (HOMO − 1) to LUMO excitations, considerably separated from the first peak. The HOMO and LUMO delocalize on the whole system, indicating that modest intramolecular electron transfer from the methylthio and amino groups on the pyrimidine ring (HOMO) to the phenyl moiety (LUMO) is expected upon S_0_→S_1_ transition. The transition character is also rationalized with the transition dipole moment directed along the long molecular axis. The electron density difference between HOMO and LUMO is shown in Figure 2; this reflects the balanced electron redistribution between the pyrimidine and phenyl moieties.

**Table 1:** Experimental and computed absorption maxima (λ_max_) for **3a**–**g**, **6**, and **7**.

No.	λ_max_ (exp.)^a^	λ_max_ (calc.)^b^

**3a**	341	335
**3b**	282	329
**3c**	341	339
**3d**	343	350
**3e**	344	371
**3f**	322	322
**3g**	322	347
**6**	361	351
**7**	342	328

^a^measured in dichloromethane, excluding **3b** (in ethanol). ^b^TD-DFT(B3LYP)/6-31+G(d,p)//DFT(B3LYP)/6-311G(d,p).

### Solid-state fluorescence

Tris(8-hydroxyquinolinato)aluminium (Alq_3_) was used as the standard for fluorescence spectrum measurements [17]. We analyzed the solid-state fluorescence emission spectra of **3a**–**g**, **6** and **7** at room temperature. The fluorescence maxima (λ_em,max_) and relative fluorescence intensities (RI) of these compounds are listed in Table 2. The λ_em,max_ values of **3a** and **3c**–**g** were in the range 430–462 nm. The fluorescence of the *N*-unsubstituted diamino compound **3b** exhibited a large bathochromic shift, and green fluorescence was observed (λ_em,max_ = 529 nm). Compound **3a**, which has an amino group at the 2-position in the pyrimidine ring, showed stronger fluorescence than did compound **3g**, which has a methylsulfanyl group. This result was in agreement with the previous findings that an amino group at the 2-position of an indenopyrimidine influences the fluorescence intensity [6]. 2,4-Diaminopyrimidine derivatives (**3b**–**e**) also showed stronger fluorescence than did **3g**. Compound **3c**, which has an amino group at the 2-position and a benzylamino group at the 4-position of the pyrimidine ring, showed the strongest fluorescence (RI = 1.95). This value was larger than those of indenopyrimidine derivatives (RI = 0.01–0.73). We speculated that the effect of the π–π-electron conjugated system in the sulfonyl group of benzothienopyrimidine 5,5-dioxide is stronger than that of the carbonyl group of the indeno pyrimidines.

**Table 2:** Solid-state-fluorescence data for **3a**–**g**, **6**, and **7**.

No.	λ_max_ (nm)	λ _em,max_ (nm)	SS^a^	Rl^b^

**3a**	341	462	118	1.16
**3b**	282	529	180	0.23
**3c**	341	435	90	1.95
**3d**	343	448	102	0.99
**3e**	344	444	95	0.58
**3f**	322	430	80	0.42
**3g**	322	430	80	0.07
**6**	361	555	218	0.84
**7**	342	425	76	0.16

^a^Stokes shift; emission - excitation in solid state. ^b^Relative intensity of fluorescence in solid states, using Alq_3_ as the standard compound.

Benzo[4,5]thieno[3,2-*d*]pyrimidine derivative **6** and benzo[4,5]furo[3,2-*d*]pyrimidine derivative **7** showed different emission properties from those of **3a**–**g**. Compound **6** exhibited a large bathochromic shift compared with that of the benzo[4,5]thieno[3,2-*d*]pyrimidine 5,5-dioxides **3a** and **3c**–**g**, and relatively strong fluorescence. In contrast, the weakly fluorescent compound **7** exhibited a hypsochromic shift relative to **3a**–**g**. These results presumably indicate that hetero atoms incorporated in the ring moieties strongly influence the solid-state-fluorescent properties.

Solid-state fluorescence is strongly influenced by intermolecular steric hindrance. In general, the fluorescence intensity can be enhanced by minimizing intermolecular interactions through the introduction of bulky substituents. Compound **3c**, which has a bulky benzylamino moiety shows the strongest fluorescence of these molecules (Figure 2). The emission from **3e** with a bulky phenylamino moiety, however, is relatively weak. This contradiction reflects the complex emission mechanism, which is driven not only by intermolecular stacking effects but also by the electronic structures. The weak fluorescence of **7** is assumed to be dominated by the inductive effect of the methoxy group, rather than by stacking effects.

**Figure 3:**
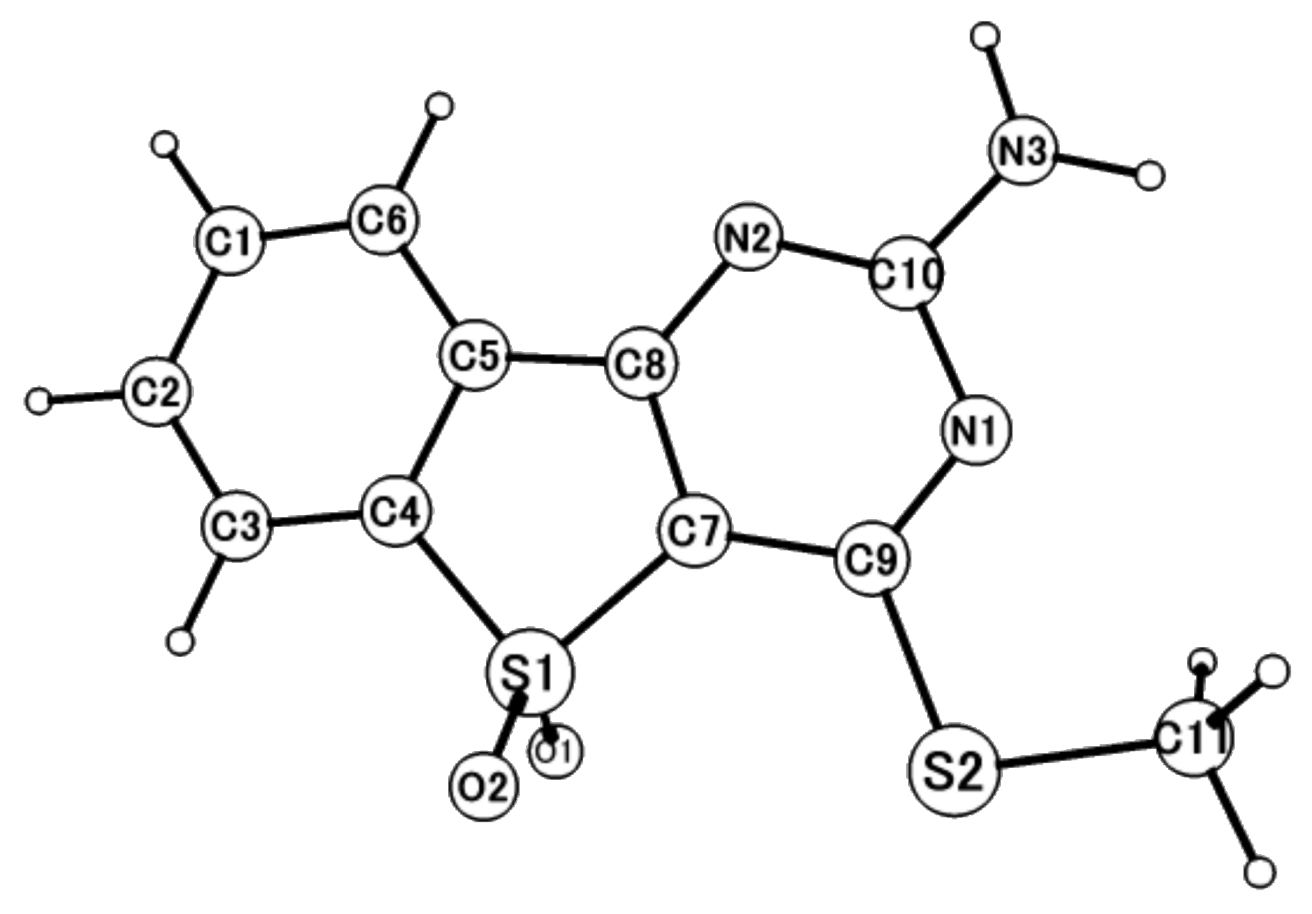
Schematic diagram of **3a**.

To elucidate the S_1_ nature of the compounds in question, we carried out ab initio molecular-orbital calculations, focused on **3a** (Figure 3). The computed key bond lengths are shown in Table 3. Comparison of the S_0_ (DFT) and S_1_ (CIS) bond lengths shows that the S_0_→S_1_ (dominant HOMO→LUMO) transition is reflected in the bond-length variations. The C4–C5 bond, with a bonding lobe in S_0_ but an antibonding one in S_1_, is significantly lengthened, by 0.045 Å, as a result of the S_0_→S_1_ transition. In contrast, the C5–C8 bond, with an antibonding lobe in S_0_ but a bonding one in S1, is considerably shortened, by 0.081 Å, on S_0_→S_1_ excitation. No significant deviation from planarity was observed in the fused-ring structure associated with the S_0_→S_1_ excitation. The geometrical discrepancies between DFT and CAS for S_0_ and CIS and CAS for S_1_ indicate that the significant roles of dynamic electron correlations are inadequate in the CAS method, which consistently predicted longer bond lengths. These disagreements could be resolved by geometric optimizations using MS-CASPT2 including dynamic correlation, but this is not feasible in the present study owing to the huge computational burden.

**Table 3:** Key bond lengths of **3a** in the S_0_ and S_1_ states.

No.	S_0_		S_1_	
	DFT^a^ [Å]	CAS^b^ [Å]	CIS^c^ [Å]	CAS^b^ [Å]

C4–C5	1.399	1.439	1.444	1.437
S1–C4	1.806	1.891	1.745	1.885
S1–C7	1.790	1.886	1.755	1.874
N2–C8	1.331	1.416	1.345	1.430
C5–C8	1.476	1.528	1.395	1.516
C7–C8	1.399	1.461	1.433	1.414
C7–C9	1.404	1.461	1.423	1.496
C9–N1	1.335	1.433	1.295	1.383
N3–C10	1.353	1.471	1.336	1.442
S2–C9	1.763	1.917	1.747	1.905
S2–C11	1.824	1.946	1.807	1.956

^a^DFT(B3LYP)/6-311G(d,p). ^b^CASSCF(10, 10)/ANO-S-MB. ^c^CIS/6-311G(d,p).

The theoretical fluorescence λ_max_ values of **3a**, obtained using several quantum-chemical methods, are summarized in Table 4. The CIS-predicted peak shows a significant blue-shift relative to the experimental value; this is well known, and is a result of the lack of multi-excitation character. The TDDFT peaks partially improve the gap, with the CIS optimized geometry for the S_1_ state not being of trustworthy quality. The CASSCF peak, in contrast, overshoots the experimental peak. The best prediction was obtained by using the most elaborate method, MS-CASPT2, which can take the majority of the electronic correlations into account. The MS-CASPT2 result indicates that the excitation character includes significant contributions from the HOMO→LUMO double excitation. This implies that the predictive abilities of CIS and other single-configuration-referenced methods, which cannot handle multi-excitation features, are limited.

**Table 4:** The computed first intense fluorescence maxima for **3a**.

	CIS^a^	TD^b^	CAS^c^	MS-CASPT2^d^	Exp. (in solid)

λ_max_ (oscillator strength)	291 (0.294)	391 (0.081)	530 (0.12 × 10^−3^)	498 (0.12 × 10^−3^)	462
excitation	H→L (0.66)	H→L (0.70)	(H)^2^ →(L)^2^ (0.66)	(H)^2^ →(L)^2^ (0.58)	

^a^TD(B3LYP)/6-31G+(d,p)//CIS/6-311G(d,p). ^b^TD(B3LYP)/6-31G+(d,p)//CIS/6-311G(d,p). ^c^CAS(10,8)/ANO-S//CAS(10,10)ANO-S-MB. ^d^MS-CASPT2(10,8)/ANO-S//CAS(10,10)/ANO-S-MB.

## Conclusion

New solid-state fluorescent compounds, benzo[4,5]thieno[3,2-*d*]pyrimidine 5,5-dioxides (**3a**–**g**), were synthesized in good yields by a convenient one-pot reaction of 2-[bis(methylsulfanyl)methylene]benzo[*b*]thiophene-3(2*H*)-one 1,1-dioxide (**1a**) with guanidine carbonate (**2a**) or (*S*)-methylisothiourea sulfate (**2b**) under reflux in pyridine. Benzo[4,5]thieno[3,2-*d*]pyrimidine derivative **6** and benzo[4,5]furo[3,2-*d*]pyrimidine derivative **7** were also synthesized by one-pot reactions between **2a** and **1b** or **1c**, under the same conditions. The products **6** and **7** showed solid-state fluorescence. Compound **3c**, which has an amino group at the 2-position and a benzylamino group at the 4-position of the pyrimidine ring, showed the strongest solid-state fluorescence. These results indicated that heteroatoms in the ring moieties have a strong influence on the solid-state emissions. The absorption and emission spectra of the compounds were computationally analyzed by means of a series of ab initio quantum-chemical calculations. The theoretical analyses quantitatively reproduced the S_0_→S_1_ and S_1_→S_0_ transitions. The associated HOMO/LUMO distributions and the transition characters were also elucidated theoretically.

## Experimental

### General

Identifications of compounds and measurements of properties were carried out by general procedures employing the following equipment: All melting points were determined with a Mitamura Riken Kogyo Mel-Temp apparatus or a Laboratory Devices Mel-Temp II apparatus and were uncorrected. Infrared (IR) spectra were recorded in potassium bromide pellets on a JASCO 810 or Shimazu IR-460 spectrometer. Ultraviolet (UV) absorption spectra were determined in 95% ethanol on a Hitachi 323 spectrometer. Fluorescence spectra were determined on a Shimazu RF-1500. Nuclear-magnetic-resonance (NMR) spectra were obtained on Gemini 300NMR (300 MHz) and 500NMR (500 MHz) spectrometers with tetramethylsilane as an internal standard. Mass spectra (MS) were recorded on a JOEL DX-303 mass spectrometer. Microanalyses were performed by H. Mazume on a Yanaco M-5 at Nagasaki University. All chemicals were reagent grade and used without further purification unless otherwise specified.

### Method of measurement of fluorescence

A powder sample of the subject compound was heaped in the tray. After the sample was covered with a quartz plate, this part was fixed in the fluorescence spectrometer. After fixing the fluorescence wavelength, the excitation spectrum was determined by scanning with the fluorescence wavelength. Similarly, the fluorescence spectrum was obtained after scanning with the excitation wavelength. After obtaining these results, the excitation wavelength was decided and the fluorescence spectrum was measured. The relative intensity of fluorescence was determined by using Alq_3_ as the standard sample. The fluorescence of the standard sample and all subject compounds was measured at 272 nm excitation.

### Synthesis

**2-Amino-4-(methylsulfanyl)benzo[4,5]thieno[3,2-*d*]pyrimidine 5,5-dioxide (3a):** A mixture of 2-[bis(methylsulfanyl)methylene]benzo[*b*]thiophene-3(2*H*)-one 1,1-dioxide (**1a**; 1.43 g, 5.0 mmol), guanidine carbonate (**2a**; 0.90 g, 5.0 mmol) and pyridine (10 mL) was stirred for 5 h under reflux. The reaction mixture was poured into ice water (100 mL). The colorless crystals that appeared were collected by filtration and washed with methanol (30 mL) to give **3a** (1.28 g, 89%) as colorless needles. An analytical sample was recrystallized from a mixture of toluene and methanol to give colorless needles. Mp 294–296 °C; IR (KBr, cm^−1^) ν: 3409, 3332, 3219, 1650, 1546, 1520, 1145; UV (CH_2_Cl_2_) λ_max_, nm (log ε): 341 (4.15), 396 (3.99), 461 (3.77); UV (EtOH) λ_max_, nm (log ε): 252 (4.43), 297 (3.91), 307 (4.04), 366 (3.92), 376 (3.93); ^1^H NMR (300 MHz, DMSO-*d*_6_) δ 2.64 (s, 3H, SMe), 7.86 (m, 2H, 7-H, 8-H), 7.95–8.04 (m, 4H, NH_2_, 6-H, 9-H); ^13^C NMR (100 MHz, DMSO-*d*_6_) δ 11.0, 114.4, 121.5, 122.9, 129.6, 134.3, 134.4, 140.3, 158.5, 164.1, 165.2; MS *m*/*z* (% relative intensity): 280 (M^+^ + 1, 17), 279 (M^+^, 100), 262 (29), 215 (36), 188 (30), 114 (21); HRMS: [M^+^] calcd for C_11_H_9_N_3_O_2_S_2_, 279.0136; found, 279.0121; Anal. calcd for C_11_H_9_N_3_O_2_S_2_: C, 47.31; H, 3.23; N, 15.05; found: C, 47.46; H, 3.25; N, 15.08.

**2-(Diaminomethylene)benzo[*b*]thiophene-3(2*H*)-one 1,1-dioxide (4):** A mixture of **1a** (1.43 g, 5.0 mmol), ammonia solution (28%, 5 mL) and methanol (10 mL) was heated under reflux for 30 min. After cooling, water and methanol were evaporated. The residue was collected, washed with water and methanol, and crystallized from methanol to give 0.98 g (4.4 mmol, 88%) of colorless needles. Mp 312–314 °C; MS *m*/*z* (% relative intensity): 225 (M^+^ + 1, 18), 224 (M^+^, 100), 159 (11), 153 (24), 104 (11), 76 (13); Anal. calcd for C_9_H_8_N_2_O_3_S: C, 48.21; H, 3.60; N, 12.49; found: C, 48.27; H 3.42; N, 12.51.

**2,4-Diaminobenzo[4,5]thieno[3,2-*d*]pyrimidine 5,5-dioxide (3b): Method A**: A mixture of 0.30 g (1.2 mmol) of **3a** and ammonium hydroxide (28%, 20 mL) in a mini-autoclave (50 mL) was heated at 200 °C for 3 h. After cooling, the precipitate that appeared was collected by filtration and washed with water to give pale yellow needles (0.14 g, 0.6 mmol, 47%). Mp 315–318 °C (dec.). **Method B**: A mixture of **4** (0.30 g, 1.2 mmol), **2a** (0.50 g, 3.0 mmol) and pyridine (10 mL) was refluxed for 7 h. After cooling, the reaction mixture was poured into water (50 mL). The precipitate that appeared was collected by filtration to give pale yellow needles (0.12 g, 0.5 mmol, 41%). Mp 320–322 °C (dec.). An analytical sample was recrystallized from methanol to give pale yellow needles. Mp 320–322 °C (dec.); IR (KBr, cm^−1^) ν: 3418 (NH), 3319 (NH), 3138 (br NH), 1666 (CO), 1563, 1501, 1402, 1232; UV (EtOH) λ_max_, nm (log ε): 274 (3.64), 282 (3.68); ^1^H NMR (300 MHz, DMSO-*d*_6_) δ 7.30 (br s, 2H, NH_2_), 7.81 (m, 2H, 7-H, 8-H), 7.92–7.98 (m, 2H, 5-H, 6-H); ^13^C NMR (100 MHz, DMSO-*d*_6_) δ 100.8, 120.9, 122.3, 130.8, 133.3, 133.8, 140.5, 156.8, 159.7, 165.5; MS *m*/*z* (% relative intensity): 249 (M^+^ + 1, 17), 248 (M^+^, 100), 203 (20), 200 (33), 136 (33); HRMS: [M^+^] calcd for C_10_H_8_N_4_O_2_S, 248.0368; found, 248.0359; Anal. calcd for C_10_H_8_N_4_O_2_S: C, 48.38; H, 3.25; N, 22.57; found: C, 48.27; H 3.22; N 22.51.

**2-Amino-4-benzylaminobenzo[4,5]thieno[3,2-*d*]pyrimidine 5,5-dioxide (3c):** A mixture of **1a** (0.57 g, 2.0 mmol), benzylamine (**5a**; 0.25 g, 2.3 mmol), **2a** (0.27 g, 1.5 mmol) and pyridine (10 mL) was stirred for 5 h under reflux. The reaction mixture was poured into ice water (100 mL). The colorless crystals that appeared were collected by filtration and washed with methanol (10 mL) to give colorless needles (0.62 g, 92%). An analytical sample was recrystallized with a mixture of toluene and methanol to give colorless needles. Mp 221–222 °C; IR (KBr, cm^−1^) ν: 3406, 3347, 3233, 1654, 1558, 1462, 1283, 1142; UV (CH_2_Cl_2_) λ_max_, nm (log ε): 258 (4.62), 332 (4.06), 341 (4.08), 397 (3.99), 463 (3.77); ^1^H NMR (300 MHz, CDCl_3_) δ 4.66 (d, *J* = 5.6 Hz, 2H, N-CH_2_-), 5.31 (br s, 2H, NH_2_), 5.64 (br s, 1H, NH), 7.28–7.38 (m, 5H, phenyl-H), 7.68 (m, 2H, 7-H, 8-H), 7.82 (m, 1H, 9-H), 7.99 (m, 1H, 5-H); ^13^C NMR (100 MHz, DMSO-*d*_6_) δ 42.9, 101.2, 120.9, 122.3, 126.7, 127.5, 128.2, 130.7, 133.3, 133.8, 139.5, 140.3, 155.5, 159.3 165.3; MS *m*/*z* (% relative intensity): 339 (M^+^ + 1, 21), 338 (M^+^, 100), 321 (62), 304 (25), 303 (44), 201 (23), 91 (47); Anal. calcd for C_17_H_14_N_4_O_2_S: C, 60.34; H, 4.17; N, 16.56; found: C, 60.35; H, 4.18; N, 16.51.

**2-Amino-4-piperidinylbenzo[4,5]thieno[3,2-*d*]pyrimidine 5,5-dioxide (3d):** This compound (0.32 g, 1.0 mmol) was prepared in 51% yield from **1a** (0.57 g, 2.0 mmol), piperidine (**5b**; 0.34 g, 4.0 mmol), and **2a** (0.30 g, 1.7 mmol) in a similar procedure to that described for the synthesis of **3c**. An analytical sample was recrystallized from a mixture of toluene and methanol to give colorless needles. Mp 224–226 °C; IR (KBr, cm^−1^) ν: 3480, 3447, 3329, 3213, 2942, 1633, 1558, 1282, 993, 551; UV (CH_2_Cl_2_) λ_max_, nm (log ε): 343 (4.16), 397 (4.03); ^1^H NMR (300 MHz, CDCl_3_) δ 1.74 (m, 6H, -(CH_2_)_3_-), 3.91 (m, 4H, N-CH_2_-), 5.11 (br s, 2H, NH_2_), 7.66 (m, 2H, 7-H, 8-H), 7.81 (m, 1H, 9-H), 8.00 (m, 1H, 6-H); ^13^C NMR (100 MHz, DMSO-*d*_6_): 24.1, 25.7, 25.7, 46.8, 46.8, 102.2, 120.8, 122.3, 130.6, 13.5, 133.9, 138.6, 156.1, 160.9, 164.08; MS *m*/*z* (% relative intensity): 317 (M^+^ + 1, 10), 316 (M^+^, 53), 299 (100), 282 (23), 127 (12), 84 (11); Anal. calcd for C_15_H_16_N_4_O_2_S: C, 56.94; H, 5.10; N, 17.71; found: C, 56.79; H, 5.11; N, 17.56.

**2-Amino-4-phenylaminobenzo[4,5]thieno[3,2-*d*]pyrimidine 5,5-dioxide (3e):** This compound (0.56 g, 1.7 mmol) was prepared in 86% yield from **1a** (0.57 g, 2.0 mmol), aniline (**5c**; 0.33 g, 3.5 mmol), and **2a** (0.30 g, 1.7 mmol) in a similar procedure to that described for the synthesis of **3c**. An analytical sample was recrystallized from a mixture of toluene and methanol to give colorless needles. Mp 252–255 °C; IR (KBr, cm^−1^) ν: 3346 (NH), 3192 (NH), 1651, 1602, 1551, 1292, 1143; UV (CH_2_Cl_2_) λ_max_, nm (log ε): 271 (4.65), 344 (4.15), 396 (3.99), 461 (3.77); ^1^H NMR (500 MHz, CDCl_3_) δ 7.15 (m, 2H, phenyl-H), 7.34 (m, 2H, phenyl-H), 7.40 (br s, 1H, NH), 7.49 (br s, 1H, NH), 7.69 (d, *J* = 7.3 Hz, 2H, phenyl-H), 7.83–7.86 (m, 2H, 7-H, 8-H), 7.97–8.03 (m, 2H, 6-H, 9-H); ^13^C NMR (100 MHz, DMSO-*d*_6_) δ 102.2, 121.0, 122.3, 123.9, 124.0, 124.3, 128.2, 128.3, 130.5, 133.6, 134.0, 138.2, 140.4, 154.3, 160.1, 165.1; MS *m*/*z* (% relative intensity): 326 (M^+^ + 2, 11), 325 (M^+^ + 1, 35), 324 (M^+^, 100), 323 (79), 260 (14), 259 (62), 91 (15); Anal. calcd for C_16_H_12_N_4_O_2_S: C, 59.25; H, 3.73; N, 17.27; found: C, 59.58; H, 3.95; N, 17.25.

**2-Amino-4-methoxybenzo[4,5]thieno[3,2-*d*]pyrimidine 5,5-dioxide (3f):** A mixture of **1a** (0.57 g, 2.0 mmol), **2a** (0.36 g, 2.0 mmol), and methanol (10 mL) was refluxed for 1 h. After cooling, the precipitate that appeared was collected by filtration to give colorless crystals (0.29 g, 1.1 mmol) in 55% yield. An analytical sample was recrystallized from toluene to give colorless needles. Mp 298–302 °C; IR (KBr, cm^−1^) ν: 3476, 3399, 3343, 3222, 1654, 1551, 1374, 1301, 1158; UV (CH_2_Cl_2_) λ_max_, nm (log ε): 253 (4.75), 322 (4.10), 397 (4.00), 462 (3.79); ^1^H NMR (300 MHz, CDCl_3_) δ 4.04 (s, 3H, OMe), 7.83–7.88 (m, 2H, 7-H, 8-H), 7.94 (br s, 1H, NH), 7.95–8.03 (m, 2H, 6-H, 9-H), 8.04 (br s, 1H, NH); ^13^C NMR (100 MHz, DMSO-*d*_6_) δ 54.2, 103.4, 121.4, 122.6, 129.8, 134.1, 134.4, 140.7, 161.5, 164.2, 166.1; MS *m*/*z* (% relative intensity): 264 (M^+^ + 1, 15), 263 (M^+^, 100), 262 (12), 246 (12), 220 (18), 152 (16), 136 (15); Anal. calcd for C_11_H_9_N_3_O_3_S, C, 50.16; H, 3.45; N, 15.96; found: C, 50.18; H, 3.41; N, 15.91.

**2,4-Bis(methylsulfanyl)benzo[4,5]thieno[3,2-*d*]pyrimidine 5,5-dioxide (3g):** This compound (0.54 g, 1.8 mmol) was obtained in 87% yield from **1a** (0.57 g, 2.0 mmol), *S*-methylisothiourea sulfate (**2b**; 0.56 g, 2.0 mmol), and pyridine (10 mL) in a manner similar to that described for the synthesis of **3a**. An analytical sample was recrystallized from toluene to give colorless needles. Mp 233–234 °C; IR (KBr, cm^−1^) ν: 2929, 1513, 1461, 1306, 1153, 869; UV (CH_2_Cl_2_) λ_max_, nm (log ε): 253 (4.75), 322 (4.10), 397 (4.00), 462 (3.79); UV (EtOH) λ_max_, nm (log ε): 208 (3.94), 275 (4.28), 345 (3.35); ^1^H NMR (300 MHz, CDCl_3_) δ 2.68 (s, 3H, SMe), 2.74 (s, 3H, SMe), 7.74 (m, 2H, 7-H, 8-H), 7.88 (m, 1H, 9-H), 8.15 (m, 1H, 6-H); ^13^C NMR (100 MHz, CDCl_3_) δ 11.7, 14.2, 121.0, 122.0, 123.8, 128.8, 135.0, 135.1, 139.3, 156.3, 164.5, 176.7; MS *m*/*z* (% relative intensity): 311 (M^+^ + 1, 17), 310 (M^+^, 100), 295 (11), 293 (18), 249 (15), 246 (11), 173 (12), 136 (12), 114 (12); Anal. calcd for C_12_H_10_N_2_O_2_S_3_: C, 46.43; H, 3.25; N, 9.02; found: C, 46.45; H, 3.20; N, 8.96.

**2-Amino-4-(methylsulfanyl)benzo[4,5]thieno[3,2-*d*]pyrimidine (6):** This compound (0.92 g, 3.7 mmol) was prepared in 74% yield from **1b** (1.27 g, 5.0 mmol) and **2a** (1.0 g, 5.6 mmol) in a manner similar to that described for the synthesis of **3a**. An analytical sample was recrystallized from a mixture of toluene and methanol to give pale yellow needles. Mp 191–193 °C; IR (KBr, cm^−1^) ν: 3487 (NH), 3313 (NH), 3186 (NH), 1630, 1524, 755, 722; UV (CH_2_Cl_2_) λ_max_, nm (log ε): 249 (4.79), 294 (4.37), 304 (4.42), 361 (4.34), 372 (4.34), 462 (3.79); ^1^H NMR (300 MHz, CDCl_3_) δ 2.69 (s, 3H, SMe), 6.88 (br s, 2H, NH_2_), 7.55 (m, 1H, 8-H), 7.67 (m, 1H, 7-H), 8.06 (d, *J* = 7.8 Hz, 1H, 9-H), 8.19 (d, *J* = 8.3 Hz, 1H 6-H); ^13^C NMR (100 MHz, CDCl_3_) δ 11.5, 115.8, 123.4, 123.9, 125.2, 130.1, 133.0, 140.2, 156.8, 161.5, 163.1; MS *m*/*z* (% relative intensity): 249 (M^+^ + 2, 45), 248 (M^+^ + 1, 75), 247 (M^+^, 100), 246 (75), 202 (39), 201 (54), 200 (33), 187 (39), 146 (54), 114 (21); Anal. calcd for C_11_H_9_N_3_S_2_: C, 53.42; H, 3.67; N, 16.99; found: C, 53.56; H, 3.64; N, 16.93.

**2-Amino-4-methylsulfanyl-7-methoxybenzo[4,5]furo[3,2-*d*]pyrimidine (7):** This compound (0.41 g, 1.6 mmol) was prepared in 65% yield from **1c** (0.64 g, 2.4 mmol) and **2a** (0.33 g, 1.8 mmol) in a manner similar to that described for the synthesis of **3a**. An analytical sample was recrystallized from a mixture of toluene and methanol to give colorless needles: Mp 203–204 °C; IR (KBr, cm^−1^) ν: 3470 (NH), 3179 (NH), 1627, 1572, 1432, 1366, 1255; UV (CH_2_Cl_2_) λ_max_, nm (log ε): 306 (4.37), 342 (4.40), 397 (3.99), 462 (3.77); ^1^H NMR (300 MHz, CDCl_3_) δ 2.66 (s, 3H, SMe), 3.90 (s, 3H, OMe), 4.97 (br s, 2H, NH_2_), 6.97 (dd, *J* = 2.0, 8.8 Hz, 1H, 8-H), 7.05 (d, *J* = 2.0 Hz, 1H, 6-H), 7.90 (1H, d, *J* = 8.8 Hz, 9-H); ^13^C NMR (100 MHz, CDCl_3_) 11.4, 55.8, 96.5, 112.8, 114.8, 122.4, 140.9, 148.1, 152.2, 159.4, 159.5, 162.7; MS *m*/*z* (% relative intensity): 263 (M^+^ + 2, 15), 262 (M^+^ + 1, 43), 261 (M^+^, 100), 260 (79), 246 (18), 216 (15), 215 (22), 214 (12), 201 (19); Anal. calcd for C_12_H_11_N_3_O_2_S: C, 55.16; H, 4.24; N, 16.08; found: C, 55.08; H, 4.32; N, 16.11.
